# Multitarget Therapeutic Strategies for Chagas Disease: Natural Compounds, Antimicrobial Peptides, and Cell-Based Immunomodulation

**DOI:** 10.3390/idr18040065

**Published:** 2026-06-30

**Authors:** Ana María Fernández-Presas, Katia Jarquín-Yáñez, Adolfo Cruz-Reséndiz, Oscar Rodríguez-Lima, Jaime Zamora-Chimal, Blanca Esther Blancas-Luciano

**Affiliations:** 1 Department of Microbiology and Parasitology, Faculty of Medicine, Universidad Nacional Autónoma de México, Mexico City 04510, Mexico; presas@unam.mx (A.M.F.-P.); acruz@facmed.unam.mx (A.C.-R.); orodriguez@facmed.unam.mx (O.R.-L.); 2 Department of Cellular and Tissue Biology, Faculty of Medicine, Universidad Nacional Autónoma de México, Mexico City 04510, Mexico; jy.katy@facmed.unam.mx; 3 Experimental Medicine Research Unit, Faculty of Medicine, Universidad Nacional Autónoma de México, Hospital General de México “Dr. Eduardo Liceaga”, Mexico City 06726, Mexico

**Keywords:** *Trypanosoma cruzi*, antiparasitic therapy, natural compounds

## Abstract

Chagas disease, caused by *Trypanosoma cruzi*, remains a major public health problem in Latin America and an emerging global health concern due to population mobility. Although benznidazole and nifurtimox remain the only approved antiparasitic drugs, their limited efficacy in chronic infection, prolonged treatment regimens, frequent adverse effects, and variable activity across parasite strains highlight the need for new therapeutic strategies. In addition, the pathogenesis of chronic Chagas disease is driven not only by parasite persistence but also by immune-mediated tissue damage, particularly in chronic Chagas cardiomyopathy. In this review, we examine emerging therapeutic approaches that extend beyond conventional trypanocidal chemotherapy, with emphasis on natural products, antimicrobial peptides, and cell-based immunomodulatory strategies. Plant compounds and essential oils have shown antiparasitic activity through mechanisms including oxidative stress induction, membrane disruption, interference with sterol biosynthesis, and mitochondrial dysfunction, while some extracts also modulate host immune responses. Antimicrobial peptides display dual potential by directly damaging parasite membranes and organelles or by reshaping infection-associated inflammatory responses. In parallel, cell-based therapies such as mesenchymal stromal cells, tolerogenic dendritic cells, and bone marrow-derived cells have demonstrated promising cardioprotective and immunoregulatory effects in experimental chronic Chagas disease. Collectively, these approaches support a multitarget therapeutic framework in which parasite-directed and host-directed interventions may complement each other. Further mechanistic studies, standardization, and translational validation will be essential to advance these candidates toward clinically useful therapies for Chagas disease.

## 1. Introduction

Chagas disease, caused by the kinetoplastid parasite *Trypanosoma cruzi*, remains one of the most important neglected tropical diseases and a persistent global public health challenge despite decades of control efforts [1]. It is estimated that more than 7 million people are currently infected worldwide, with the highest burden concentrated in Latin America, where transmission remains endemic in at least 21 countries [2]. In addition to ongoing vectorial transmission, increased population mobility has led to a growing number of autochthonous and imported cases in non-endemic regions, including North America, Europe, Asia, and Australia, further reinforcing the global relevance of Chagas disease [3]. The disease disproportionately affects socioeconomically vulnerable populations and is associated with substantial long-term healthcare and socioeconomic costs, largely driven by chronic cardiac and digestive complications [1,2].

Transmission occurs primarily through hematophagous triatomine insects of the subfamily Triatominae; however, congenital, transfusional, and oral routes also contribute to disease spread [4]. Following infection, *T. cruzi* establishes a complex life cycle involving both insect vectors and vertebrate hosts. Infective trypomastigotes invade nucleated host cells, escape from the parasitophorous vacuole, and differentiate into intracellular amastigotes, which replicate within the cytoplasm [5]. This intracellular lifestyle promotes long-term parasite persistence across multiple tissues and represents a central biological barrier to achieving sterile cure [6].

Clinically, *T. cruzi* infection progresses from an acute phase, often asymptomatic or mildly symptomatic, to a prolonged indeterminate stage characterized by low-level parasite persistence in the absence of overt disease [3,7]. Approximately 30–40% of infected individuals subsequently develop chronic Chagas disease, most commonly chronic Chagas cardiomyopathy, which constitutes the most severe manifestation and a leading cause of disease-related mortality [3]. Importantly, disease progression is primarily driven by persistent infection and host-mediated inflammatory responses rather than by high levels of circulating parasites [7].

Current treatment relies almost exclusively on the nitroheterocyclic drugs benznidazole and nifurtimox, which require activation by parasite nitroreductases to exert trypanocidal activity [8]. Although these agents achieve relatively high cure rates during acute infection, their efficacy in chronic disease remains limited [9]. Moreover, prolonged treatment regimens and frequent adverse effects—including dermatological reactions, gastrointestinal disturbances, and neurological toxicity—significantly compromise treatment adherence and clinical outcomes [10].

In addition, parasite genetic diversity represents a major obstacle to effective chemotherapy. *T. cruzi* is classified into multiple discrete typing units (DTUs), which differ in tissue tropism, virulence, and drug susceptibility [11,12]. Resistance to benznidazole and other investigational compounds has been associated with reduced nitroreductase expression and enhanced antioxidant defenses [13]. Together, intracellular persistence, immune-mediated tissue damage, and parasite genetic heterogeneity highlight critical limitations of current therapies and underscore the need for novel strategies capable of targeting both parasite survival mechanisms and host–parasite interactions.

Taken together, the persistent global burden of Chagas disease, its chronic and progressive clinical course, and the limited efficacy of available treatments emphasize the urgent need for alternative and complementary therapeutic approaches. In this narrative review, we provide a critical overview of emerging multitarget therapeutic strategies against *T. cruzi*, focusing on natural compounds, antimicrobial peptides, and immunomodulatory approaches. Increasing attention has therefore been directed toward antimicrobial peptides and other natural compounds with multitarget activity, as well as immunomodulatory strategies aimed at restoring host immune balance and combination therapies designed to enhance efficacy while reducing drug-associated toxicity [14,15,16,17]. In this review, we critically examine these emerging approaches, with emphasis on their mechanisms of action and therapeutic potential to overcome current limitations in Chagas disease management.

## 2. Search Strategy and Selection Criteria

A literature search was performed using major scientific databases, including PubMed, Scopus, and Web of Science. Search terms consisted of combinations of “*Trypanosoma cruzi*,” “Chagas disease,” “natural compounds,” “essential oils,” “antimicrobial peptides,” and “cell-based therapy,” using Boolean operators (AND/OR).

Articles published in English were considered, with priority given to recent experimental (in vitro and in vivo) and preclinical studies. Relevant review articles were also included to provide contextual background. Studies were selected based on their relevance to mechanisms of action, immunomodulatory effects, and multitarget therapeutic strategies against *T. cruzi*.

No formal systematic review protocol was followed, and study selection was based on the authors’ critical assessment of the literature.

## 3. Conventional Treatment for Chagas Disease

**Figure 1:**
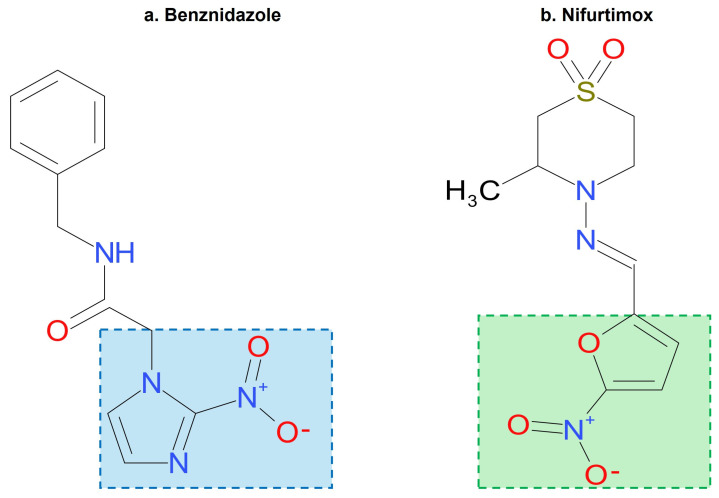
Chemical structures of benznidazole and nifurtimox. (**a**) Benznidazole, (**b**) Nifurtimox. The nitroimidazole moiety in benznidazole and the nitrofuran moiety in nifurtimox are highlighted to indicate the nitroheterocyclic scaffolds associated with their prodrug activation and trypanocidal activity. Chemical structures were drawn using the Chem4Word (Version 3.3.15, Chem4Word Team, Cambridge, UK) add-in for Microsoft Word.

Therapeutic options for Chagas disease remain limited, relying primarily on two nitro-derivative compounds developed more than 50 years ago: nifurtimox [3-methyl-4-(nitrofurfurylideneamino)tetrahydro-4H-1,4-thiazine-1,1-dioxide] (Nfx, Lampit^®^), a nitrofuran derivative, and benznidazole [N-benzyl-2-nitroimidazole acetamide] (Bnz, Rochagan^®^, Roche, Basel, Switzerland), a nitroimidazole derivative. Both nitroheterocyclic drugs contain a nitro group attached to either a furan or an imidazole ring, respectively [11,18,19]. The chemical structures of both drugs are shown in Figure 1, highlighting the nitroheterocyclic scaffold that underlies their prodrug behavior. They act as prodrugs that require activation by parasite nitroreductases (NTRs), a key step for their trypanocidal activity. This process begins with the reduction of the nitro group to a nitro anion radical catalyzed by parasite NTR enzymes [8].

Two main classes of NTRs have been identified in *Trypanosoma* species. Type II NTRs are flavin-containing enzymes that initiate redox cycling, generating unstable nitro radicals through interaction with molecular oxygen [20]. In contrast, Type I NTRs are flavin mononucleotide (FMN)-dependent, oxygen-insensitive enzymes that catalyze two-electron reduction reactions. This group includes enzymes such as prostaglandin F2α synthase-like reductases, as well as mitochondrial NTRs that utilize NADH or NADPH as electron donors [8,21]. The resulting reactive intermediates induce oxidative stress and cause DNA damage, ultimately impairing essential cellular processes [8,22].

This dependence on metabolic activation represents a critical vulnerability of current nitroheterocyclic therapy, since alterations in this step can directly affect drug susceptibility and contribute to resistance. In *T. cruzi*, reduced nitroreductase activity, point mutations or premature stop codons in the TcNTR gene, and broader metabolic adaptations have been associated with decreased benznidazole activation and resistance [23]. Moreover, naturally resistant strains may exhibit metabolic plasticity involving enzymes such as aldo-keto reductases and alcohol dehydrogenases, together with compensatory changes in pathways related to redox balance and energy metabolism [24].

Additional resistance mechanisms include increased expression of antioxidant enzymes such as superoxide dismutases (SOD), which protect parasites from oxidative damage, as well as mutations in DNA repair pathways and alterations in ergosterol biosynthesis—the latter also implicated in resistance to posaconazole [25,26]. This variability is further complicated by the broad genetic diversity of *T. cruzi*, which is classified into six DTUs, TcI–TcVI, together with the bat-associated lineage TcBat. Differences among DTUs in geographic distribution, tissue tropism, metabolic activity, oxidative stress responses, immune evasion, and intracellular persistence may contribute to heterogeneous drug susceptibility and therapeutic outcomes [27,28]. In addition, the complex genome of *T. cruzi* encodes large multigene families of surface proteins, including trans-sialidases, mucins, mucin-associated surface proteins (MASPs), and GP63 proteases, which participate in host–parasite interactions and may further influence parasite persistence and treatment response [29]. Thus, benznidazole resistance and therapeutic failure should be interpreted as multifactorial processes shaped by molecular resistance mechanisms, parasite genetic background, and host–parasite interactions.

Although both drugs are approved for trypanosomiasis, benznidazole exhibits a more favorable safety and tolerability profile and is generally considered the first-line treatment. In the United States, benznidazole is approved by the U.S. Food and Drug Administration (FDA) for pediatric patients 2–12 years of age, whereas nifurtimox is approved for pediatric patients from birth to younger than 18 years of age weighing at least 2.5 kg [30,31].

Despite these regulatory indications, the clinical use of both drugs remains constrained by prolonged treatment duration, (60–90 days) adverse events, and adherence issues. Benznidazole and nifurtimox achieve cure rates of approximately 60–85% during the acute phase, as determined by parasite clearance [32]. However, their clinical performance remains far from the characteristics expected for an optimal antichagasic therapy, which should achieve parasitological cure in both acute and chronic stages, require only a single or limited number of doses, and lack teratogenicity or severe adverse effects [33].

Although benznidazole has been associated with long-term cure rates of up to 75% after 27 years of follow-up, in specific pediatric cohorts, its clinical use in adults is limited by frequent adverse reactions, treatment discontinuation, and reduced efficacy during chronic infection [34]. Adverse effects associated with benznidazole have been reported in 48% of treated patients, commonly presenting as hypersensitivity reactions (dermatitis), gastrointestinal intolerance, and neurological symptoms, while hematological toxicity is rare but potentially severe [8,32,35]. The most reported adverse effects of nifurtimox include anorexia, weight loss, and neuropsychiatric manifestations such as irritability and sleep disturbances [36]. Comparative studies conducted in Latin America have reported wide variations in treatment completion rates, ranging from 58.6% to 88.4% for benznidazole and dropping from 25% to 96.3% for nifurtimox, directly compromising therapeutic outcomes [34,35].

Despite relatively high cure rates during acute infection, treatment effectiveness in chronic Chagas disease is substantially lower in adult patients, particularly in endemic settings, and does not significantly prevent disease progression in individuals with established cardiomyopathy [37]. Importantly, the limited efficacy observed in chronic infection reflects the inability of current drugs to eliminate intracellular parasite reservoirs and to adequately modulate host immune responses. Indeed, benznidazole and nifurtimox act primarily as trypanocidal agents and were not designed to simultaneously address the multiple pathological components of chronic Chagas disease, including parasite persistence, sustained inflammation, tissue remodeling, and progressive fibrosis. These limitations reinforce the need for therapeutic strategies capable of combining direct antiparasitic activity with immunomodulatory, anti-inflammatory, or tissue-protective effects.

These limitations provide the clinical and biological rationale for recent efforts aimed at optimizing existing regimens and identifying new drug candidates.

## 4. Recent Clinical Trials and Emerging Drug Candidates

Recent clinical trials and drug-development efforts have produced mixed results, with a clear shift from the search for a single superior monotherapy toward treatment optimization and combination-based strategies. In the BENEFIT trial, benznidazole reduced the detection of *T. cruzi* DNA in blood from patients with established Chagas cardiomyopathy, but this parasitological effect did not translate into a significant reduction in cardiac clinical deterioration after 5 years of follow-up [38]. This finding highlights a central limitation of current therapy: parasite suppression alone may be insufficient once chronic inflammatory and fibrotic cardiac damage is established.

In this context, recent studies have explored shorter or lower-dose benznidazole regimens to preserve antiparasitic activity while improving tolerability and adherence. The BENDITA phase 2 trial evaluated alternative benznidazole regimens, alone or combined with fosravuconazole, in adults with chronic indeterminate Chagas disease [39]. Similarly, the BETTY trial was designed to compare a short, low-dose benznidazole regimen with the standard regimen in *T. cruzi*-seropositive postpartum women, aiming to reduce adverse effects and improve treatment completion [40].

By contrast, several emerging or repurposed monotherapies have shown limited efficacy or translational bottlenecks. Fexinidazole, evaluated in the FEXI-12 phase 2 trial for chronic indeterminate Chagas disease, showed limited efficacy, with sustained PCR negativity achieved in only a small proportion of participants and safety concerns including delayed-onset neutropenia and transient liver enzyme elevations [41]. Similarly, posaconazole failed to achieve sustained parasitemia clearance comparable to benznidazole, while E1224/fosravuconazole produced only non-sustained parasite clearance in chronic infection [42,43,44].

These limitations have renewed interest in drug repositioning and combination therapies. Preclinical studies suggest that metronidazole, ivermectin, and chloroquine may enhance anti-*T. cruzi* activity when combined with benznidazole or nifurtimox, potentially improving parasite clearance while reducing exposure to standard nitroheterocyclic drugs [45,46]. However, these combinations remain experimental and require rigorous pharmacokinetic, toxicological, and clinical validation.

Overall, recent evidence suggests that future Chagas disease therapy may depend less on replacing benznidazole with a single superior monotherapy and more on designing optimized, stage-specific, and multitarget regimens. Such strategies should aim to reduce parasite burden, improve tolerability, reach intracellular reservoirs, limit chronic inflammation, and prevent progressive tissue remodeling. This therapeutic gap supports the exploration of complementary parasite-directed and host-directed approaches, including natural compounds, antimicrobial peptides, immunomodulatory strategies, and rational combination therapies.

## 5. Emerging Therapeutic Strategies Against *T. cruzi*

### 5.1. Natural Products

#### 5.1.1. Plant- and Microalgae-Derived Extracts

Phytotherapy represents one of the earliest therapeutic practices worldwide, and more than 21,000 plant species are currently used medicinally according to the World Health Organization [47]. Natural products constitute a major source of drug discovery, with approximately 60% of approved drugs derived from natural product metabolites or their derivatives [48]. In this framework, numerous plant-derived compounds have demonstrated biological activity relevant to Chagas disease [49]. Representative plant- and microalgae-derived extracts evaluated against *T. cruzi*, including their parasite stages, IC_50_ values, cytotoxicity, and proposed mechanisms, are summarized in Table 1.

Among these, alkaloids from the *Amaryllidaceae* family have shown antiparasitic activity against *Plasmodium falciparum*, *Leishmania donovani*, and *T. cruzi* [50,51]. In line with these findings, extracts from Amaryllidaceae species such as *Crinum erubescens* and *Rhodophiala andicola* exhibited significant anti-*T. cruzi* activity, with half-maximal inhibitory concentration (IC_50_) values of approximately 6–10 ppm. Notably, these extracts were active against the intracellular amastigote stage while maintaining low cytotoxicity toward HepG2 cells (selectivity index, SI > 20) [50]. The evaluation of intracellular amastigotes is particularly relevant because this stage more closely reflects mammalian infection than epimastigote-based screening. Nevertheless, the absence of pharmacokinetic data and in vivo efficacy limits the ability to infer whether these active concentrations are achievable under physiological conditions.

Several medicinal plants traditionally used in Mexico for the treatment of parasitic infections have also been evaluated. Methanolic extracts of *Eryngium heterophyllum*, *Haematoxylum brasiletto*, *Marrubium vulgare*, and *Schinus molle* demonstrated strong inhibition of parasite growth (88–100%), with IC_50_ values of 11.24, 7.92, 22.66, and 16.31 μg/mL, respectively [52]. Similarly, *Bidens pilosa* methanolic extract and its fractions inhibited more than 90% of both epimastigote and trypomastigote stages at approximately 800 μg/mL, without significant cytotoxicity in Vero cells [53]. However, the wide variability in effective concentrations reflects the heterogeneity of plant-derived preparations, including differences in extraction solvent, plant part, phytochemical profile, and batch composition. In many cases, the lack of compound isolation, quantitative chemical standardization, cytotoxicity comparison in mammalian cells, and in vivo validation remains a major limitation, making it difficult to distinguish true lead candidates from preliminary screening hits.

Beyond in vitro observations, only a limited number of studies have provided in vivo evidence together with mechanistic insights into the antitrypanosomal activity of plant-derived metabolites. Oral administration of the hydroethanolic extract of *Aristeguietia glutinosa* (Asteraceae) at 50 mg/kg, as well as treatment with its most active component (+)-15-hydroxy-7-labden-17-al at 30 mg/kg, significantly reduced parasitemia in a murine model of acute *T. cruzi* infection. These effects were associated with inhibition of parasite mitochondrial dehydrogenases and sterol biosynthesis pathways [54]. Although these findings represent an important advance beyond in vitro screening, further studies are required to define oral bioavailability, metabolic stability, dose–response relationships, tissue distribution, and safety margins before considering these metabolites as translational candidates.

In addition to direct antiparasitic effects, increasing evidence indicates that certain plant-derived extracts can modulate host immune responses, a feature of relevance in Chagas disease. Extracts of *Clethra fimbriata* demonstrated in vitro trypanocidal activity against epimastigotes, trypomastigotes, and amastigotes of *T. cruzi*. The hexane extract exhibited IC_50_/EC_50_ values of 153.9 ± 29.5 μg/mL, 39.3 ± 7.2 μg/mL, and 45.6 ± 10.5 μg/mL, respectively (Figure 1). Cell death analysis using Annexin V/PI staining revealed that the extract predominantly induced early apoptosis in epimastigotes, whereas in trypomastigotes it increased the frequency of early apoptosis, late apoptosis, and necrosis [55]. Moreover, treatment was associated with low cytotoxicity and modest immunomodulatory effects on CD4^+^ and CD8^+^ T cells, including increased production of IFN-γ and TNF-α, as well as de novo expression of granzyme B and perforin. These responses may contribute to parasite clearance through inducible nitric oxide synthase (iNOS) activation or cytotoxic activity against infected cells [55,56].

**Figure 2:**
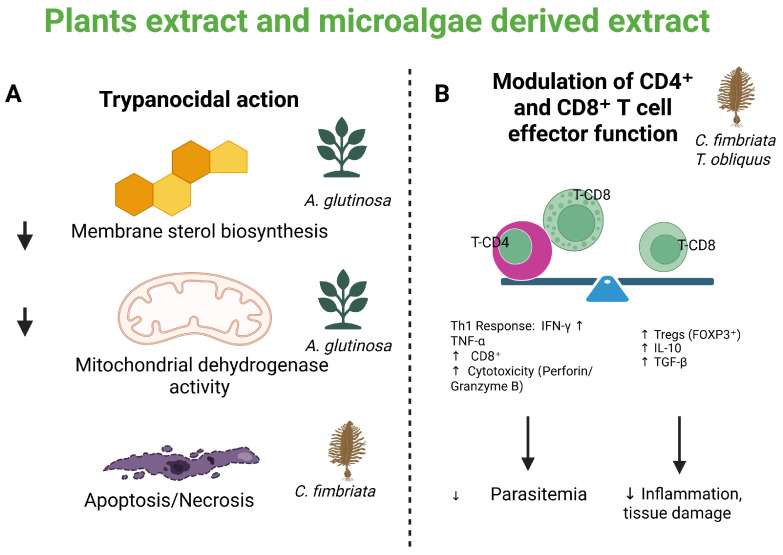
Dual mechanisms of plant-derived and microalgae compounds against *Trypanosoma cruzi*: direct antiparasitic activity and host immune modulation. (**A**) The plant-derived labdane diterpenoid (+)-15-hydroxy-7-labden-17-al, isolated from Aristeguietia glutinosa, exerts direct antiparasitic effects against *T. cruzi*, including inhibition of parasite sterol biosynthesis and mitochondrial dehydrogenase activity, leading to reduced parasite viability. Additionally, other phytotherapeutic agents, such as *Clethra fimbriata*, have been reported to induce apoptotic and necrotic pathways in the parasite. (**B**) Beyond their direct parasiticidal activity, plant-derived compounds can also modulate host immune responses by influencing the balance between Th1 effector mechanisms and regulatory pathways. For example, extracts of *C. fimbriata* have been associated with enhanced Th1 responses, including increased IFN-γ and TNF-α production and cytotoxic CD8^+^ T-cell activity. In contrast, extracts from microalgae such as *Tetradesmus obliquus* may promote regulatory responses, characterized by increased IL-10 production, potentially contributing to the establishment of a regulatory immune microenvironment. These combined effects ultimately influence infection outcome by shaping the balance between parasite clearance and host tissue damage. Arrows indicate the proposed direction of the biological effects and pathway relationships. Created with BioRender.com (Version 2026, BioRender, Toronto, ON, Canada; Agreement number: AU29WCM0YO; https://BioRender.com/rsxwowb, accessed on 12 February 2026).

Additional evidence of immunomodulation has been reported for other natural sources. Aqueous extracts of the microalgae *Chlorella vulgaris* and *Tetradesmus obliquus* reduced IFN-γ levels, contributing to attenuation of the inflammatory response. Furthermore, *T. obliquus* induced a modest increase in TNF-α production while promoting the secretion of the regulatory cytokine IL-10 [57] (Figure 2). Whether these effects involve the expansion or functional activation of regulatory T cells remains unclear, but such mechanisms could be relevant for limiting tissue damage during infection (Figure 2). In this context, evidence from other protozoan models may also help identify natural-product scaffolds with future relevance for Chagas disease. Prenylated flavonoids from *Mundulea sericea* have shown antiprotozoal activity against *Plasmodium falciparum* and *Leishmania donovani*, with low or negligible cytotoxicity and, in some cases, induction of nitric oxide production in murine cells [58]. Although these compounds have not yet been directly evaluated against *T. cruzi*, their combined antiparasitic and immunomodulatory properties support their consideration as candidates for future anti-*T. cruzi* screening. Given the dual contribution of parasite persistence and host immune responses to Chagas disease pathogenesis, the immunological effects of these extracts warrant careful evaluation to determine whether they confer protection or exacerbate pathology. However, the therapeutic significance of these immune changes remains difficult to predict without integrated infection models, since enhanced pro-inflammatory or cytotoxic responses may contribute to parasite control but may also exacerbate tissue injury depending on disease stage and parasite burden.

Overall, current evidence indicates a progression from initial in vitro antiparasitic screening to the emerging recognition of in vivo efficacy and immunomodulatory properties. While these findings support the multifaceted therapeutic potential of plant-derived extracts, they also emphasize the need for rigorous characterization of active compounds, standardized formulations, and integrated studies addressing both antiparasitic activity and host immune modulation. Importantly, future studies should prioritize reproducible chemical fingerprints, activity-guided fractionation, clinically relevant parasite stages, pharmacokinetic profiling, and toxicity assessment in validated in vivo models. Advancing these candidates toward translational application will require coordinated chemical, parasitological, and immunological approaches.

#### 5.1.2. Essential Oils

Essential oils (EOs) are volatile, hydrophobic liquids extracted from plants and composed primarily of terpenes and terpenoids, along with phenylpropanoids. Although EOs are plant-derived products and may be considered plant extracts in a broad sense, they are discussed separately in this review because they represent chemically distinct volatile mixtures, commonly obtained by distillation and typically characterized by gas chromatography (GC) or gas chromatography–mass spectrometry (GC-MS) analysis [59]. They exhibit a broad spectrum of biological activities, including antimicrobial, anti-inflammatory, antiulcer, antioxidant, antiviral, and antiparasitic effects. Their pharmacological properties are strongly influenced by plant chemotype, extraction method, and chemical stability, as these factors critically determine essential oil composition and bioactivity [60,61,62]. This compositional variability represents one of the main obstacles for reproducibility, since oils obtained from the same plant species may differ substantially in the relative abundance of active constituents depending on geography, seasonality, plant organ, extraction conditions, and storage.

**Figure 3:**
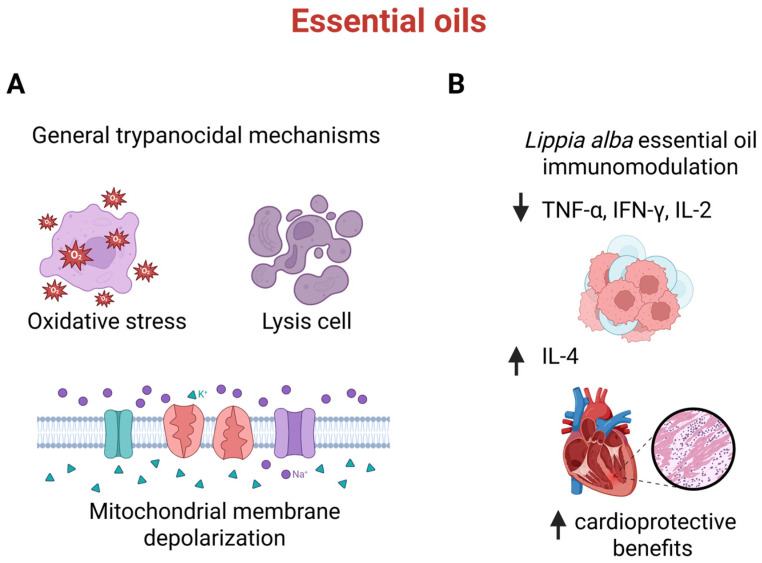
Antiparasitic and immunomodulatory effects of essential oils during *Trypanosoma cruzi* infection (**A**) Essential oils exert direct antiparasitic activity both in vitro and in vivo by inducing oxidative stress, leading to parasite lysis. Due to their high lipophilicity and ability to permeate cellular membranes, these compounds can act intracellularly, particularly by disrupting mitochondrial function and promoting mitochondrial membrane depolarization. (**B**) In addition to their direct parasiticidal effects, certain essential oils display immunomodulatory properties. In vitro studies using *T. cruzi*–infected macrophages have shown that fractions derived from *Lippia alba* essential oil, alone or in combination with benznidazole, reduce pro-inflammatory mediators such as IFN-γ and TNF-α while increasing IL-4 production. This immunoregulatory profile may contribute to the cardioprotective effects and improved histopathological architecture observed in chronic experimental Chagas disease. Arrows indicate the proposed direction of the biological effects and pathway relationships. Created with BioRender.com (Version 2026, BioRender, Toronto, ON, Canada; Agreement number: WN29WCMHC0; https://BioRender.com/4mextbs, accessed on 12 February 2026).

The antimicrobial activity of essential oils involves multiple complementary mechanisms, including disruption of membrane integrity leading to increased permeability and leakage of intracellular contents, impairment of energy metabolism through ATP depletion and respiratory inhibition, induction of oxidative stress via reactive oxygen species (ROS) generation, and interference with quorum sensing and biofilm formation, ultimately resulting in cell death [63]. In the context of parasitic infections, these effects have been associated with oxidative stress induction, enhanced lipid peroxidation, and subsequent membrane destabilization, leading to severe cellular damage in parasites (Figure 3) [64]. Nevertheless, many of these mechanisms have been extrapolated from antibacterial or antifungal models, and their direct contribution to parasite killing in *T. cruzi* remains insufficiently validated for several essential oils.

Clove essential oil (*Syzygium aromaticum* L.) has demonstrated potent in vitro activity against epimastigote and bloodstream trypomastigote forms of *T. cruzi*. Using steam-distilled oils characterized by GC, and GC-MS, clove EO exhibited IC_50_ values of 99.5 µg/mL for epimastigotes and 57.5 µg/mL for trypomastigotes, representing the highest activity among the essential oils evaluated. Treatment with clove EO and its major constituent, eugenol, induced pronounced ultrastructural alterations, predominantly affecting the parasite nucleus, as revealed by scanning and transmission electron microscopy [65].

Notably, *Cinnamomum verum* essential oil exhibits greater antiparasitic potency. This oil reduced epimastigote viability with an IC_50_ of 24.13 µg/mL after 24 h of exposure, while metacyclic trypomastigotes showed even higher susceptibility (IC_50_ = 5.05 µg/mL). Importantly, significant activity was also observed against intracellular amastigotes within infected Vero cells (IC_50_ = 20 µg/mL), supporting its relevance against clinically pertinent parasite stages and highlighting stage-dependent differences in susceptibility [66]. Oregano essential oil also demonstrated in vitro trypanocidal activity, inhibiting epimastigote growth (IC_50_/24 h = 175 μg/mL) and inducing lysis of bloodstream trypomastigotes (IC_50_/24 h = 115 μg/mL) after 24 h of exposure [67]. However, the interpretation of these IC_50_ values should be accompanied by selectivity indices, cytotoxicity profiles, and exposure conditions, since hydrophobic compounds may display assay-dependent effects related to solubility, volatility, or interactions with culture media [21,22]. A comparative summary of essential oils and their major bioactive components evaluated against *T. cruzi*, including parasite stages, IC_50_ values, cytotoxicity or selectivity data, and proposed mechanisms of action, is presented in Table 2.

Evidence from in vivo studies further supports the therapeutic potential of essential oils. Clove and ginger essential oils reduced parasitemia and parasite burden in experimental *T. cruzi* infection, although a reduction in mortality was observed only in animals treated with ginger essential oil [68]. Fractions derived from *Lippia alba* essential oils, rich in citral, caryophyllene oxide, and limonene, exhibited trypanocidal efficacy comparable to benznidazole and provided additional cardioprotective effects in a chronic experimental model, including attenuation of cardiac dilation and improved histopathological architecture [69]. In vitro studies using *T. cruzi*-infected macrophages further demonstrated that these fractions, alone or in combination with benznidazole, exert immunomodulatory effects characterized by reduced production of pro-inflammatory mediators such as IFN-γ and TNF-α, along with increased IL-4 levels [70]. These findings may partially explain the cardioprotective effects observed in vivo and highlight the potential of essential oils to modulate host–parasite interactions. Despite these encouraging findings, in vivo interpretation remains constrained by limited information on formulation, systemic exposure, metabolic stability, tissue distribution, and dose standardization. These parameters are particularly important for essential oils because their volatility, hydrophobicity, and complex chemical composition may affect bioavailability and therapeutic reproducibility [71,72].

Taken together, essential oils exhibit relevant trypanocidal activity and, in selected cases, cardioprotective and immunomodulatory effects in experimental Chagas disease; however, variability in chemical composition remains a major barrier to reproducibility across studies. Their progression toward clinical application is further limited by the lack of standardized chemotypes, defined active constituents, pharmacokinetic characterization, long-term toxicity studies, and formulation strategies capable of overcoming poor aqueous solubility and volatility. Advancing these compounds will require the use of chemically defined fractions, reproducible methodologies, and rational integration with existing therapies to improve efficacy and translational potential.

#### 5.1.3. Mushroom- and Fungal-Derived Natural Products

Fungi, including macrofungi and endophytic species, represent an underexplored reservoir of structurally diverse secondary metabolites with potential relevance for Chagas disease drug discovery. Compared with plant-derived extracts and essential oils, the evidence supporting mushroom- and fungal-derived natural products against *T. cruzi* remains limited; however, available studies suggest that these organisms can provide bioactive scaffolds with direct trypanocidal activity, target-specific effects, or immunomodulatory properties. Representative mushroom- and fungal-derived extracts/metabolites evaluated against *T. cruzi*, including their target parasite stages, IC_50_ values, cytotoxicity or selectivity data, and proposed mechanisms of action, are summarized in Table 3.

Among mushroom-derived compounds, ergosterol isolated from the basidiomycete *Pleurotus salmoneostramineus* showed activity against *T. cruzi* trypomastigotes, with an IC_50_ of 51.3 µg/mL. Mechanistically, ergosterol induced plasma membrane permeabilization and mitochondrial membrane depolarization without increasing reactive oxygen species (ROS) levels in epimastigotes, a phenotype often associated with the triggering of an apoptosis-like cell death pathway. Importantly, no cytotoxicity was observed in mammalian cells at concentrations up to 200 µg/mL, suggesting a preliminary selectivity profile [73]. Similarly, bioassay-guided fractionation of *Lentinus strigosus* led to the identification of hypnophilin, a fungal sesquiterpenoid with activity against intracellular amastigotes and inhibitory effects on *T. cruzi* trypanothione reductase, an essential enzyme involved in parasite redox homeostasis [74]. In addition, the meroterpenoid austin, produced during co-culture of the endophytic fungi *Talaromyces purpurogenus* H4 and *Phanerochaete* sp. H2, showed trypanocidal activity against epimastigotes, illustrating how fungal co-culture strategies can activate secondary metabolism and uncover otherwise silent bioactive metabolites [75].

Natural products active against related trypanosomatids may also guide future Chagas disease research focusing on fungal biodiversity. For example, a carbohydrate fraction from the edible mushroom *Astraeus hygrometricus* reduced *Leishmania donovani* burden in vitro and in vivo while promoting proinflammatory cytokine production, Toll-like receptor expression, and macrophage-associated protective responses [77]. Although these findings cannot be directly extrapolated to *T. cruzi*, they support the further exploration of fungal-derived fractions as potential sources of antiparasitic and immunomodulatory molecules for future Chagas disease studies.

Despite these promising findings, mushroom- and fungal-derived natural products remain at an early preclinical stage. Most evidence is restricted to dose–response curves obtained from in vitro assays using isolated metabolites or crude extracts, and data on pharmacokinetics, formulation, toxicity, in vivo efficacy, and reproducibility are still scarce [78,79]. Moreover, because immune-mediated parasite control differs among trypanosomatid infections, future studies should not only assess the direct trypanocidal activity of these products, but also determine whether their immunomodulatory effects favor parasite clearance without promoting inflammatory tissue damage. Therefore, these compounds should be interpreted as promising bioactive scaffolds rather than advanced therapeutic candidates.

### 5.2. Antimicrobial Peptides

Antimicrobial peptides (AMPs) are typically short, cationic molecules characterized by an amphipathic structure, generally ranging from 10 to 50 amino acids in length and exhibiting net positive charges that facilitate interactions with microbial membranes; however, substantial variability exists in their size, charge, and mechanisms of action [80,81]. AMPs display potent and broad-spectrum antimicrobial activity against bacteria, yeasts, fungi, viruses, and parasites. Their activity is primarily driven by electrostatic interactions with negatively charged microbial membrane surfaces, leading to membrane disruption through pore formation or peptide aggregation, ultimately resulting in osmotic lysis [82,83]. Despite these advantages, the therapeutic development of AMPs is often limited by proteolytic degradation, short systemic half-life, potential hemolytic or immunogenic effects, manufacturing costs, and the need for delivery systems or structural optimization to improve stability and selectivity.

**Figure 4:**
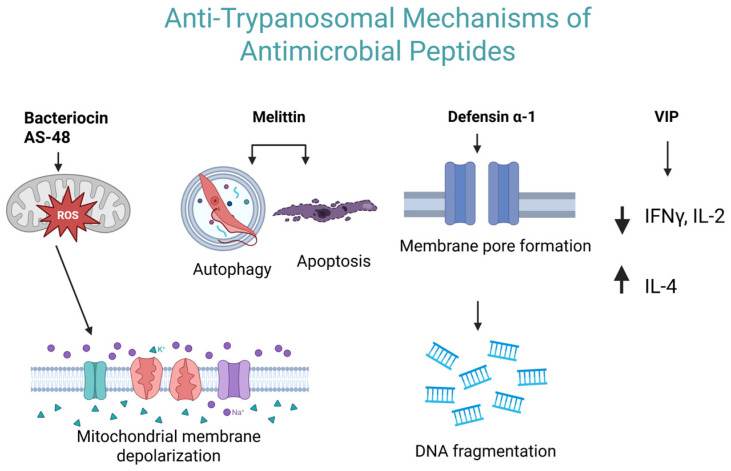
Anti-*Trypanosoma* mechanisms mediated by antimicrobial and immunomodulatory peptides. Antimicrobial peptides (AMPs) exert antiparasitic activity through multiple complementary mechanisms. The bacteriocin AS-48 induces intracellular reactive oxygen species (ROS) overproduction, leading to mitochondrial membrane depolarization, bioenergetic failure, and parasite death. Melittin exhibits stage-dependent effects, inducing autophagy in epimastigotes and apoptosis in trypomastigotes. Human α-defensin 1 promotes membrane pore formation, resulting in loss of membrane integrity and subsequent nuclear and mitochondrial DNA fragmentation. Beyond direct parasiticidal activity, vasoactive intestinal peptide (VIP) modulates host immune responses during acute *Trypanosoma cruzi* infection by attenuating Th1 responses (reduced IFN-γ and IL-2) and promoting IL-4 production, thereby reshaping the inflammatory environment. Arrows indicate the proposed direction of the biological effects and pathway relationships. Created with BioRender.com. (Version 2026, BioRender, Toronto, ON, Canada; Agreement number: UY29WCORZ9; https://BioRender.com/t85pfjn, accessed on 12 February 2026).

In protozoan parasites, AMPs have been shown to alter membrane fluidity and interfere with the function of membrane-associated proteins. In *T. cruzi*, these peptides exert microbicidal effects through the activation of multiple cell death pathways, including plasma membrane permeabilization, mitochondrial dysfunction, and parasite lysis (Figure 4) [84]. Representative antimicrobial and immunomodulatory peptides evaluated in the context of *T. cruzi* infection, including their origin, target parasite stage, activity values, cytotoxicity or selectivity data, and proposed mechanisms of action, are summarized in Table 4. Adade et al. (2013) demonstrated that melittin induces autophagy in epimastigotes (IC_50_ = 2.44 ± 0.23 μg/mL), whereas in trypomastigotes it triggers apoptosis (LD_50_ = 0.14 ± 0.05 μg/mL), highlighting stage-dependent susceptibility, although the underlying molecular mechanisms remain unclear [85].

Other peptides, such as temporizin and hemocyanin-derived peptides, have been reported to induce necrotic cell death. Epimastigotes treated with temporizin at its IC_50_ (16.8 µM) exhibited mitochondrial dysfunction, nuclear alterations, and an increased number of reservosomes [86]. In contrast, hemocyanin-derived peptides promote reactive oxygen species (ROS) production and membrane pore formation, both hallmarks of necrotic cell death [87]. Despite these observations, the downstream signaling pathways triggered by peptide-induced damage remain only partially characterized. In addition, comparisons among peptide studies are complicated by differences in parasite stage, strain background, incubation time, peptide purity, serum conditions, and readouts used to distinguish membrane disruption, apoptosis-like death, autophagy, or necrosis.

Recent studies on cruzioseptin CZS-5 have reported potent and selective activity against *T. cruzi* epimastigotes (IC_50_ ≈ 4.7 µM), associated with membrane permeabilization via toroidal pore formation. This mechanism is supported by DNA leakage assays, ultrastructural analyses, and molecular dynamics simulations. Complementary metabolomic profiling further revealed secondary effects on glycerophospholipid metabolism, oxidative stress, and parasite energy pathways [88]. Similarly, the bacteriocin AS-48, produced by *Enterococcus* species, exhibits activity against *Trypanosoma* and *Leishmania*. In *Trypanosoma brucei*, AS-48 is internalized through the flagellar pocket and demonstrates low toxicity in Vero cells along with resistance to exopeptidases. Its trypanocidal activity is associated with ROS production and mitochondrial depolarization [89]. From a translational perspective, AS-48 is particularly relevant because it combines trypanocidal activity with low cytotoxicity in uninfected Vero cells and lacks pore-forming activity in both Vero cells and human erythrocytes, suggesting a favorable selectivity profile toward parasite membranes or parasite-specific uptake pathways. In addition, its circular bacteriocin structure may represent a pharmacological advantage, since peptide cyclization is generally associated with improved conformational rigidity and greater resistance to degradation compared with linear peptides [89]. Thus, AS-48 illustrates how structural features can partially overcome some of the classical limitations of peptide-based therapeutics, although further validation in mammalian infection models and pharmacokinetic studies remains necessary.

Human α-defensin 1 displays potent trypanocidal activity against infective stages of *T. cruzi*, primarily mediated by membrane pore formation, resulting in loss of membrane integrity and subsequent nuclear and mitochondrial DNA fragmentation. This effect is concentration-dependent, antibody-blockable, and requires an intact parasite membrane potential, supporting a pore-dependent mechanism of action. Ultrastructural analyses further revealed extensive membrane disorganization and intracellular damage following peptide entry, ultimately reducing parasite infectivity in human epithelial cells [90]. In addition to their direct antiparasitic effects, some peptides exert significant immunomodulatory functions. The divergent effects of vasoactive intestinal peptide (VIP) reported across experimental and clinical studies can be explained by differences in disease stage and biological context. In murine models of acute *T. cruzi* infection, exogenous VIP acts as a therapeutic immunomodulator by attenuating Th1 responses, reducing IFN-γ and IL-2 levels, and increasing IL-4 production [91]. Although this shift does not significantly affect parasitemia, it effectively limits cardiac inflammation and tissue damage. Conversely, in patients with chronic Chagas cardiomyopathy, reduced endogenous VIP levels are associated with increased IL-17 expression and progressive cardiac dysfunction [92]. In this setting, decreased VIP reflects the loss of a regulatory mechanism, favoring sustained Th17-driven immunopathology rather than parasite control.

These findings highlight the context-dependent role of peptides in modulating host–parasite interactions. Therefore, immunomodulatory peptides should not be evaluated solely by their capacity to reduce inflammation, but also by their effects on parasite persistence, tissue remodeling, and long-term cardiac outcomes. In this context, antimicrobial peptides exhibit a wide functional spectrum in *T. cruzi* infection, encompassing direct trypanocidal activity and modulation of host immune responses. While many peptides induce parasite death through membrane disruption and mitochondrial dysfunction, others primarily influence disease outcome by shaping infection-associated immunopathology without directly affecting parasitemia. This functional duality highlights their therapeutic potential, but also underscores the need for deeper mechanistic characterization, standardization, in vivo validation, and pharmacological optimization. Although peptide-based candidates are attractive because of their potency and selectivity, their therapeutic development is commonly constrained by poor in vivo stability, proteolytic degradation, limited half-life, delivery to intracellular targets, potential host–cell toxicity or immunogenicity, and production cost [47,48]. Importantly, these pharmacological barriers have not been systematically evaluated for all anti-*T. cruzi* peptides, which limits direct comparison among candidates and complicates assessment of their clinical applicability in Chagas disease.

### 5.3. Cell-Based Immunomodulatory Strategies for Chronic Chagas Cardiomyopathy

Approximately 30–40% of individuals infected with *T. cruzi* progress to the chronic phase of Chagas disease, developing cardiac, digestive, or neurological manifestations, with chronic Chagas cardiomyopathy (CCM) representing the most severe and life-threatening outcome [93]. In patients with advanced CCM, heart transplantation remains the only intervention capable of substantially improving survival and quality of life [94]. However, its clinical applicability is markedly constrained by high costs, limited donor availability [95], and the requirement for lifelong immunosuppression in the context of persistent infection, which increases the risk of *T. cruzi* reactivation [96]. Consequently, heart transplantation is feasible for only a small subset of patients, underscoring a critical unmet need for therapeutic strategies capable of modifying disease progression rather than merely replacing the failing heart.

**Figure 5:**
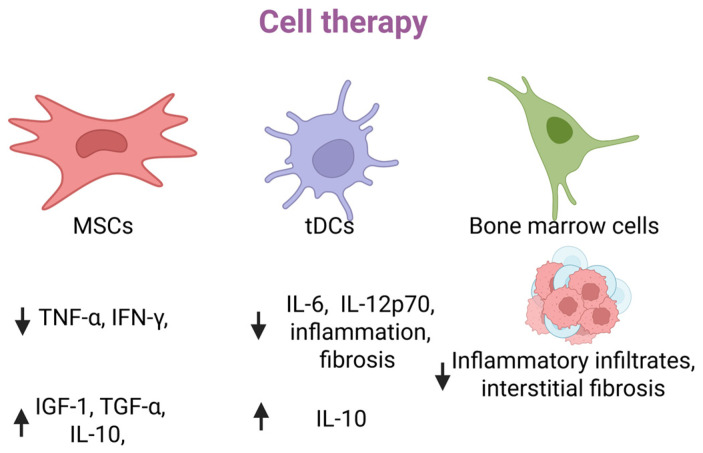
Immunomodulatory and regenerative mechanisms of cell-based therapies in chronic Chagas cardiomyopathy. Mesenchymal stem cells (MSCs) modulate the cardiac inflammatory microenvironment and promote tissue repair by reducing pro-inflammatory cytokines and increasing anti-inflammatory mediators such as IL-10. Tolerogenic dendritic cells enhance immune regulation by increasing IL-10 production and promoting the expansion of regulatory T cells (Tregs), thereby limiting myocardial inflammation and fibrosis. Bone marrow mononuclear cells attenuate inflammatory infiltrates and interstitial fibrosis in experimental Chagas cardiomyopathy. Arrows indicate the proposed direction of the biological effects and pathway relationships. Created with BioRender.com (Version 2026, BioRender, Toronto, ON, Canada; Agreement number: WC29WCPP1Y; https://BioRender.com/t85pfjn, accessed on 12 February 2026).

Within this framework, cell-based therapies have emerged as promising disease-modifying approaches for heart failure, aiming to promote myocardial regeneration, improve cardiac function, and reverse adverse ventricular remodeling [97,98]. Unlike conventional pharmacological therapies, which have limited capacity to reverse the structural myocardial damage associated with chronic Chagas cardiomyopathy, regenerative strategies such as stem cell therapy have been proposed as alternative therapeutic approaches [99]. These therapies offer the possibility of intervening earlier in the disease course and may potentially delay or prevent the need for heart transplantation. Accordingly, several experimental studies in Chagas disease have explored the therapeutic potential of different cell populations, including mesenchymal stem cells (MSCs), bone marrow-derived mononuclear cells, and dendritic cells (Figure 5) [98,99]. A comparative summary of the main cell-based therapeutic strategies evaluated in experimental and clinical Chagas cardiomyopathy, including cell type, experimental model, route of administration, therapeutic effects, proposed mechanisms, and translational limitations, is presented in Table 5.

Among these strategies, MSCs have received considerable attention due to their combined regenerative and immunomodulatory properties. Accumulating evidence indicates that MSCs secrete a broad repertoire of bioactive factors capable of reshaping the inflammatory microenvironment characteristic of Chagas disease in both acute and chronic stages. These cells also promote the secretion of pro-regenerative and immunomodulatory mediators such as insulin-like growth factor-1 (IGF-1), which contribute to tissue repair and modulation of inflammatory responses in cardiac and skeletal muscle [100]. During early infection, administration of adipose tissue-derived mesenchymal stromal cells (ASCs) has been shown to reduce parasite burden, attenuate myocardial fibrosis, and prevent right ventricular dilation, effects associated with increased production of the anti-inflammatory cytokine IL-10 [101].

In chronic models of *T. cruzi* infection, MSCs isolated from cardiac tissue significantly reduced myocardial inflammatory infiltrates and downregulated TNF-α expression, while increasing TGF-β levels in cardiac tissue, supporting an immunomodulatory mechanism of action [102]. Notably, cell-tracking studies using nanoparticle-labeled MSCs demonstrated that only a small fraction of administered cells localized to the heart, whereas the majority preferentially homed to peripheral organs such as the liver, lungs, and spleen. Despite this limited cardiac engraftment, treated animals exhibited reduced ventricular dilation, supporting the concept that MSC-mediated benefits are primarily driven by indirect or paracrine mechanisms rather than direct cardiomyocyte replacement [99]. This finding is important from a translational perspective because it challenges the initial regenerative interpretation of MSC therapy and suggests that therapeutic efficacy may depend more on secreted mediators, extracellular vesicles, or immune reprogramming than on durable myocardial engraftment.

Additional studies using adipose-derived MSCs have also reported reductions in myocardial inflammation and fibrosis in chronic experimental Chagas cardiomyopathy [103]. These findings are consistent with the broadly recognized immunomodulatory and reparative properties of MSCs, which involve paracrine interactions with immune cells and the production of regulatory mediators such as TGF-β and IL-10 during tissue repair and regeneration [106]. Further enhancement of these therapeutic effects has been achieved through MSC priming strategies. MSCs engineered to overexpress granulocyte colony-stimulating factor (G-CSF) induced a more pronounced reduction in myocardial inflammation and fibrosis, accompanied by decreased levels of IFN-γ and TNF-α and a sustained increase in IL-10 production [110]. Collectively, these findings position MSC-based therapies as promising disease-modifying strategies for chronic Chagas cardiomyopathy, acting primarily through immunomodulation and attenuation of pathological cardiac remodeling. Nevertheless, priming or genetic modification strategies may also increase manufacturing complexity, regulatory requirements, batch-to-batch variability, and the need for mechanism-relevant potency assays, all of which could limit clinical scalability [30,111].

These observations highlight immunomodulation as a central mechanism underlying the therapeutic effects of MSC-based interventions and provide a conceptual bridge to strategies that more directly target immune regulation. In this context, tolerogenic dendritic cells (tDCs) have emerged as a complementary and mechanistically aligned approach to controlling immune-mediated myocardial damage in chronic Chagas cardiomyopathy. Compared with mature myeloid dendritic cells (mDCs), tDCs are characterized by reduced production of IL-6 and IL-12p70 and enhanced secretion of IL-10, resulting in impaired T-cell activation and proliferation. Functionally, tDCs promote the expansion of FoxP3^+^ regulatory T cells, reinforcing immune tolerance. Consistent with this profile, administration of tDCs in murine models of chronic *T. cruzi* infection significantly reduced myocardial inflammation and interstitial fibrosis, leading to attenuation of adverse cardiac remodeling and disease progression [104,105]. However, the clinical translation of tDC-based therapy will require defining stable tolerogenic phenotypes, reproducible generation protocols, optimal dosing schedules, and safety profiles that avoid excessive immunosuppression or impaired parasite control.

Within this same immunomodulatory paradigm, bone marrow-derived cells (BMCs) have also demonstrated significant therapeutic potential in experimental models of chagasic cardiomyopathy. In murine systems, transplanted BMCs were shown to migrate to the heart, where they markedly reduced inflammatory infiltrates and interstitial fibrosis through mechanisms associated, at least in part, with apoptosis of inflammatory cells. Importantly, these effects were sustained for up to six months following transplantation [107]. Moreover, longitudinal assessment by cardiac magnetic resonance imaging demonstrated that BMC therapy induced regression of right ventricular dilation, with structural improvements maintained for several months post-treatment, indicating a durable attenuation of adverse cardiac remodeling [112].

Building on this robust preclinical evidence, early translational efforts advanced toward clinical evaluation using autologous bone marrow-derived mononuclear cells, given their accessibility and established safety profile. Initial clinical reports of intracoronary BMC infusion in patients with advanced chagasic cardiomyopathy documented improvements in left ventricular ejection fraction, ventricular dimensions, and functional capacity shortly after treatment, supporting both feasibility and short-term therapeutic benefit. These findings were subsequently reinforced by pilot clinical studies in patients with severe heart failure due to Chagas disease, in which intracoronary administration of autologous BMCs was well tolerated and was associated with sustained improvements in cardiac function, functional class, and quality of life at mid-term follow-up [108,109]. However, the clinical evidence remains preliminary, as most studies have involved small cohorts, limited follow-up periods, heterogeneous patient populations, and variable endpoints. Larger randomized controlled trials are still required to determine whether functional improvements are sustained, clinically meaningful, and superior to optimized standard heart failure therapy.

Taken together, these experimental and early clinical findings support a unifying model in which distinct cell-based therapies MSCs, tolerogenic dendritic cells, and bone marrow derived cells, converge on the modulation of immune-driven mechanisms that sustain myocardial inflammation and fibrosis in chronic Chagas cardiomyopathy. Rather than relying on durable myocardial engraftment or direct cardiomyocyte replacement, these approaches primarily exert their therapeutic effects through paracrine signaling, immune regulation, and attenuation of pathological cardiac remodeling. This conceptual convergence provides a strong translational rationale for the development of optimized cellular strategies, including cell priming, immune conditioning, or combinatorial approaches, aimed at enhancing the durability and specificity of immunomodulatory effects. Such strategies hold promise for redefining cell therapy as a disease-modifying intervention capable of slowing or halting progression toward end-stage heart failure in patients with chronic Chagas cardiomyopathy.

Despite this strong rationale, several barriers must be addressed before these approaches can be widely translated into clinical practice. These include donor-to-donor variability, cell manufacturing and quality control, route of administration, optimal timing of intervention, durability of therapeutic effects, long-term safety, cost, regulatory complexity, and the challenge of treating patients with advanced and heterogeneous cardiac disease. Therefore, future studies should integrate standardized potency assays, clinically relevant endpoints, long-term follow-up, and randomized controlled designs to define the real therapeutic value of cell-based interventions in chronic Chagas cardiomyopathy.

## 6. Toward Integrated Therapeutic Strategies for Chagas Disease

Although natural compounds, essential oils, antimicrobial peptides, and cell-based immunomodulatory strategies expand the therapeutic landscape for Chagas disease, their development remains limited by methodological, pharmacological, and practical challenges. A major limitation across preclinical studies is the limited comparability of experimental designs. Differences in *T. cruzi* strains or discrete typing units, parasite developmental stages, treatment duration, viability assays, host–cell models, and selectivity criteria can directly influence reported IC_50_/EC_50_ values and selectivity indices [27,113,114]. As a result, findings obtained in epimastigote-based screening may not reliably predict activity against intracellular amastigotes or efficacy in mammalian infection models.

For natural products and essential oils, reproducibility remains a central challenge because biological activity is closely linked to chemical composition. Variation in botanical source, extraction procedures, chemical profile, storage conditions, and formulation can alter the relative abundance of active constituents and, consequently, the apparent trypanocidal or immunomodulatory effects [71,72,115]. Therefore, future studies should prioritize authenticated botanical material, quantitative chemical fingerprints, batch-to-batch quality control, activity-guided fractionation, and metabolomics-based approaches to better connect chemical composition with biological activity [116,117]. In parallel, pharmacokinetic and toxicological profiling should be incorporated earlier in preclinical development, particularly for candidates intended to act against intracellular amastigotes or cardiac tissue infection, where intracellular access, tissue distribution, metabolic stability, and sustained exposure are essential [6,113,118]. For essential oils, this limitation is amplified by volatility, hydrophobicity, chemical instability, and complex composition, which can affect formulation, bioavailability, dose standardization, and therapeutic reproducibility [71,119].

The therapeutic classes discussed in this review also illustrate why integrated approaches may be more realistic than isolated interventions. Plant-derived compounds and essential oils may combine direct trypanocidal activity with modulation of inflammatory or oxidative pathways; antimicrobial peptides may affect parasite membranes, mitochondria, and intracellular viability; and cell-based therapies mainly act by reshaping immune and reparative responses in damaged cardiac tissue. These complementary mechanisms suggest opportunities for rational combinations of parasite-directed, immunomodulatory, and tissue-protective strategies. However, possible synergistic, additive, or antagonistic interactions remain poorly explored and should be evaluated using standardized combination assays, clinically relevant parasite stages, and in vivo models that assess both parasite burden and host tissue outcomes. Feasibility must also be considered: antimicrobial peptides require optimization for stability, intracellular delivery, safety, and cost, whereas cell-based therapies require reproducible manufacturing, potency assays, long-term safety assessment, scalable implementation, and realistic applicability in endemic regions [120,121,122,123]. Although early clinical studies using autologous bone marrow-derived cells in advanced chagasic cardiomyopathy suggest feasibility and possible functional improvement [108,109], current evidence remains preliminary, and larger controlled studies are still required to determine whether these interventions provide durable and clinically meaningful benefit.

## 7. Conclusions

Chagas disease remains a complex and unresolved global health challenge, particularly in its chronic phase, where parasite persistence, immune-mediated tissue damage, cardiac remodeling and the genetic heterogeneity of *T. cruzi* limit the effectiveness of conventional trypanocidal therapy. Although benznidazole and nifurtimox remain the cornerstone of treatment, their reduced efficacy in chronic infection, frequent adverse effects, prolonged treatment regimens and limited ability to halt disease progression in a substantial proportion of patients underscore the urgent need for alternative and complementary therapeutic strategies.

Accumulating preclinical evidence supports the potential of antimicrobial peptides, plant-derived compounds, essential oils, and cell-based therapies as promising approaches capable of targeting multiple aspects of Chagas disease pathogenesis, including parasite survival, host immune dysregulation, chronic inflammation and tissue remodeling. These strategies offer the advantage of multitarget activity and, in some cases, immunomodulatory or regenerative effects that may complement parasite-directed chemotherapy. Rather than acting through a single therapeutic axis, these strategies may contribute to a broader multitarget framework in which direct antiparasitic activity is integrated with host-directed immunomodulatory or tissue-protective effects. This conceptual integration is particularly relevant for Chagas disease, in which parasite persistence and host-mediated pathology jointly drive long-term clinical outcomes.

However, the translational potential of these strategies remains uneven. Most available data are still restricted to in vitro studies or early experimental models, and several critical barriers persist, including methodological heterogeneity, variability in compound composition, limited pharmacokinetic and toxicological characterization, insufficient validation in clinically relevant parasite stages, and the lack of robust clinical trials. For natural products and essential oils, chemical standardization, formulation, and reproducibility remain central challenges; for antimicrobial peptides, stability, intracellular delivery, host–cell toxicity, immunogenicity, and production costs must be addressed; and for cell-based therapies, safety, scalability, regulatory requirements, long-term efficacy, and feasibility in endemic regions remain major concerns.

From a clinical perspective, advancing these emerging therapies will require the integration of parasite-targeted and host-directed strategies, supported by rigorous standardization, mechanistic validation, pharmacological profiling, and well-designed translational studies. Future research should also explore rational combinations capable of enhancing parasite clearance while limiting chronic inflammation and preventing irreversible organ damage. Ultimately, moving from isolated experimental screening toward integrated and clinically feasible therapeutic development strategies will be essential to redefine the treatment landscape of Chagas disease and address longstanding unmet needs in affected populations.

## Figures and Tables

**Table 1:** Plant- and microalgae-derived natural products evaluated against *T. cruzi*.

Species	Family	Principal Component	Strain and Target Stage	Experimental Model	Antimicrobial Activity Against *T. cruzi* (IC_50_, LC_50_)	Cytotoxicity (TC_50_, SI, CC_50_)	Action Mechanism	Reference
*Rhodophiala andicola*	Amaryllidaceae	Tazettine and other alkaloids	Tulahuen/Trypomastigote and Amastigote	In vitro	6.13 ppm–10.18 ppm (amastigote)	TC_50_ (Vero): 228.4 µg/mL, SI: 37.27; TC_50_ (HepG2): 188.1 ppm, SI: 30.7	Reduction in parasite viability linked to methylenedioxy and tertiary nitrogen motifs.	[50,51]
*Crinum erubescens*	Amaryllidaceae	Not in source	Tulahuen/Trypomastigote and Amastigote	In vitro	9.50 ppm–11.10 ppm (amastigote)	TC_50_ (Vero): 234.7 µg/mL, SI: 24.69; TC_50_ (HepG2): 678.3 µg/mL SI: 71.4	Reduction in parasite viability linked to methylenedioxy and tertiary nitrogen motifs.	[50,51]
*Haematoxylum brasiletto*	Fabaceae	Gallic acid, methyl gallate, phloroglucinol, hematoxylin	CL Brener/Epimastigote	In vitro	7.92 µg/mL	None reported	Parasite growth inhibition mediated by trypanocidal phenolic compounds.	[52]
*Eryngium heterophyllum*	Apiaceae	Not in source	CL Brener/Epimastigote	In vitro	11.24 µg/mL	None reported	Antiparasitic mechanism remains to be elucidated.	[52]
*Schinus molle* (*Peruvian Pepper*)	Anacardiaceae	α -phellandrene, β-phellandrene, p-cymene	CL Brener/Epimastigote	In vitro	16.31 µg/ML	None reported	Direct trypanocidal activity mediated by terpenes; additional repellent and insecticidal effects against *Triatoma infestans* nymphs and eggs.	[52]
*Marrubium vulgare*	Lamiaceae	Terpenes (α -eudesmol, etc.) and flavonoids (luteolin, vitexin)	CL Brener/Epimastigote	In vitro	22.66 µg/mL	None reported	Reduction in parasite viability mediated by flavonoids and terpenes, including luteolin and apigenin.	[52]
*Bidens pilosa*	Asteraceae	2-hydroxy-3-methylbenzaldehyde (50.9%), linalool (15.4%)	Brener and Nuevo Leòn/Epimastigote and Trypomastigote	In vitro	LC_50_ (epimastigote): 318 µg/mL; LC_50_ (trypomastigote): 552 µg/mL	CC_50_ (Vero): 1003 µg/mL; SI: 3.1	Phenol- and alkaloid-rich fractions impair parasite growth through metabolic disruption.	[53]
*Aristeguietia glutinosa*	Asteraceae	(+)-15-hydroxy-7-labden-17-al	CL Brener and Y strain/Epimastigote and Trypomastigote	In vitro and In vivo (Murine model)	19.6 µg/mL (Epimastigote in vitro); reduced parasitemia at 50 mg/kg b.w. (in vivo)	Non-cytotoxic against murine macrophages; well tolerated in mice	Disruption of sterol biosynthesis and mitochondrial metabolism, likely through inhibition of squalene-2,3-epoxidase and parasite dehydrogenases such as fumarate reductase	[54]
*Clethra fimbriata*	Clethraceae	Triterpenes (Ursolic acid, Betulinic acid) and Flavonoids	Y-strain (DTU II)/Epimastigote, Trypomastigote, Amastigote	In vitro	IC_50_ (epimastigote): 153.9 µg/mL; EC_50_: 39.3 µg/mL; IC_50_ (amastigote): 45.6 µg/mL	CC_50_ (Vero): >1000 µg/mL; SI (trypomastigote): 25.4; SI (amastigote): 21.9	Apoptosis-like parasite death combined with T-cell-mediated immunomodulation, including increased IFN-γ, TNF, granzyme B, and perforin production.	[55,56]
*Tetradesmus obliquus*	Chlorophyceae	Proteins, fatty acids, pigments	Y-strain/Trypomastigote	In vitro	15.08 µg/mL	CC_50_ (PBMC): 253.44 µg/mL; SI: 16.8	TNF/IL-10-mediated immunomodulation balancing parasite control and tissue protection.	[57]
*Chlorella vulgaris*	Chlorophyceae	Proteins and Polysaccharides	Y-strain/Trypomastigote	In vitro	112.1 µg/mL	CC_50_ (PBMCs): 1000 µg/mL; SI: 8.9	Decreased IFN-associated immunomodulation and parasite structural disruption	[57]

IC_50_: half-maximal inhibitory concentration; LC_50_: lethal concentration 50%; TC_50_: toxic concentration 50%; SI: selectivity index; EC_50_: Maximal effective concentration; CC_50_: half-maximal cytotoxic concentration; DTU: discrete typing unit; PBMCs: peripheral blood mononuclear cells.

**Table 2:** Essential oils and major bioactive components evaluated against *T. cruzi*.

Species Essential Oil	Family	Principal Component	Strain and Target Stage	Experimental Model	Antimicrobial Activity Against *T. cruzi* (IC_50_)	Cytotoxicity (SI or CC_50_)	Action Mechanism	Reference
*Cinnamomum verum*	Lauraceae	(E)-cinnamaldehyde and eugenol	Dm28c/epimastigotes, metacyclic trypomastigotes and amastigotes	In vitro	24.13 µg/mL (epimastigotes); 5.05 µg/mL (trypomastigotes); 20 µg/mL (amastigotes)	CC_50_: 49.4 µg/mL (Vero cells); SI: 2.05 (epimastigotes), 9.78 (trypomastigotes)	Induction of cytosolic redox imbalance through covalent interaction with parasite proteins.	[66]
Oregano essential oil (*Origanum vulgare* L.)	Labiatae	3-ciclohen-1-ol	Y strain/epimastigotes and bloodstream trypomastigotes	In vitro	175 µg/mL (epimastigotes); 115 µg/mL (trypomastigotes)	Not reported	Plasma membrane disruption and flagellar myelin-like alterations.	[67]
Clove essential oil (*Syzygium aromaticum* L.)	Myrtaceae	Eugenol	Y strain (TcII)/epimastigotes and bloodstream trypomastigotes	In vitro and In vivo (Male Swiss Mus musculus mice)	99.5 µg/mL (epimastigotes); 57.5 µg/mL (trypomastigotes)	Not reported	Nuclear ultrastructural alterations associated with reduced parasite burden and blood culture positivity.	[68]
Ginger essential oil (*Zingiber officinale*)	Not in source	α -pinene, β-pinene, zingiberene	Y strain (TcII)/bloodstream trypomastigotes	In vivo (Male Swiss Mus musculus mice)	Not reported	Not reported	Reduction in parasite burden and mortality rate in infected mice.	[68]
*Lippia alba* (OxiLim fraction mix)	Verbenaceae	Limonene, Citral, Caryophyllene oxide	338Cl2 (TcI)/amastigotes	In vivo (Wistar rats)	80% negative results in heart qPCR (after 30 doses)	No histological evidence of toxicity found in spleen, liver, kidney, lung, and colon biopsies after treatment	Cardioprotection, tissue recovery, and immunomodulation associated with apoptosis-like parasite death, mitochondrial membrane potential loss, ROS increase, reduced TNF-α/IFN-γ, and increased IL-10/IL-4.	[69]
*Lippia alba* (*ACT1 fraction*)	Verbenaceae	Limonene	*T. cruzi* Sylvio X10/1 strain(TcI)/amastigotes	In vitro	45 ± 1.7 µg/mL	CC_50_ (J774A.1 macrophages): 458 ± 4.2 μg/mL; SI: 10.1	Reduction in pro-inflammatory cytokines, antioxidant, and induction of apoptosis.	[70]

IC_50_: half-maximal inhibitory concentration; SI: selectivity index; CC_50_: half-maximal cytotoxic concentration.

**Table 3:** Mushroom- and fungal-derived extracts/metabolites evaluated against *T. cruzi*.

Fungal Source	Compound/Isolate	Strain and Target Stage	Experimental Model	Antimicrobial Activity Against *T. cruzi* (IC_50_)	Cytotoxicity (SI, CC_50_)	Action Mechanism	Reference
*Pleurotus salmoneostramineus*	Ergosterol	Y strain/Trypomastigotes	In vitro	51.3 μg/mL	CC_50_ (murine macrophages): >200 μg/mL SI: >3.9	Permeabilization of the plasma membrane and depolarization of mitochondrial membrane potential.	[73]
*Pleurotus ostreatus*	Ergosterol peroxide	Epimastigotes	In vitro	6.7 μg/mL	Not specified (approx. 8-fold more active than ergosterol).	Presence of the endoperoxide group, a known structure with biological properties.	[73]
*Lentinus strigosus*	Hypnophilin	Tulahuen strain/Intracellular amastigotes	In vitro	2.5 μM	SI: 3.56 Non-cytotoxic at 4 μM. (PBMC)	Inhibition of trypanothione reductase (TryR) via nucleophilic attack on active site thiols.	[74]
*Lentinus strigosus*	Panepoxydone	Tulahuen strain/Intracellular amastigotes	In vitro	8.7 μM	SI: 0.15 (PBMC).	Inhibition of TRyR enzyme.	[74]
*Phanerochaete* sp. *H2* & *T. purpurogenus* H4	Austin	Y strain/Epimastigotes	In vitro	36.6 μg/mL	CC_50_ (rat cardiomyoblasts (H9c2): 175.65 μg/mL, SI: 4.79	Defense or signaling response triggered by microbial stress during fungal co-culture.	[75]
Endophytic Fungus (isolate UFMGCB 508)	Crude Extract	Tulahuen strain/Amastigotes	In vitro (L929 fibroblasts; human cancer cell lines)	1 μg/mL	Mentioned as having high selective activity (L929 fibroblasts; human cancer cell lines).	Strong inhibition of TryR enzyme.	[76]

IC_50_: half-maximal inhibitory concentration; CC_50_: cytotoxic concentration 50%; SI: selectivity index; PBMC: peripheral blood mononuclear cells; TryR: trypanothione reductase.

**Table 4:** Comparative profile of antimicrobial peptides tested against *T. cruzi*.

Antimicrobial Peptides	Length	Origin	Strain and Target Stage	Experimental Model	Antimicrobial Activity Against *T. cruzi* (IC_50_, LD_50_)	Cytotoxicity (SI or CC_50_)	Proposed Mechanism	Reference
Melittin	26 residues	*Apis mellifera venom*	CL Brener clone/epimastigotes, trypomastigotes, and intracellular amastigotes	In vitro	Epimastigotes: 2.44 ± 0.23 μg/mL; Trypomastigotes (LD_50_): 0.14 ± 0.05 μg/mL; Amastigotes: 0.22 ± 0.09 μg/mL	LLC-MK2 rhesus monkey kidney epithelial cell line: CC_50_ > 5 μg/mL; SI: 35.7 (trypomastigotes), 33.3 (amastigotes)	Stage-dependent programmed cell death involving autophagy/apoptosis and mitochondrial dysfunction.	[85]
Temporizin	16 residues	Artificial hybrid (Temporin A N-terminus, Gramicidin pore-forming region, Poly-L/K C-terminus)	Y strain/epimastigotes	In vitro	IC_50_: 795 ng/mL; 855.7 ng/mL	J774 mouse macrophage-like cells: IC_50_ = 116.9 μg/mL; GH3 rat pituitary cell3: 161.1 μg/mL; Jurkat: 134.3 μg/mL; Peritoneal macrophages: 115 μg/mL	Membrane ion-channel formation associated with cytoplasmic and mitochondrial alterations, chromatin condensation, and reservosome swelling.	[86]
Temporizin-1	13 residues	Artificial hybrid (shortened form of Temporizin)	Y strain/epimastigotes	In vitro	IC_50_: 817.3 ng/mL; 887.2 ng/mL	J774: IC_50_ = 129.3 μg/mL; GH3 (rat pituitary cells): 341.9 μg/mL; Jurkat human T lymphoblast cells: 59.09 μg/mL; Peritoneal macrophages: 3.6 μg/mL	Small ion-channel formation linked to intracellular organelle damage.	[86]
Hmc364-382	19 residues	Hemocyanin derived (Penaeus monodon shrimp)	Y strain/epimastigotes, trypomastigotes, amastigotes	In vitro	Epimastigotes: 4.79 μM; Trypomastigotes: 3.62 μM; Amastigotes: 3.62 μM	LLC-MK2 rhesus monkey kidney epithelial cell line: CC_50_ > 200 μM; SI: >55.24	Necrotic parasite death associated with membrane disruption and increased ROS production.	[87]
Hmc666-678	13 residues	Hemocyanin derived (*Penaeus monodon shrimp*)	Y strain/epimastigotes and trypomastigotes	In vitro	Epimastigotes: 4.01 μM; Trypomastigotes: 4.41 μM	LLC-MK2 rhesus monkey kidney epithelial cell line: *CC*_50_ > 200 μM; SI: >45.35	Hydrophobicity-driven membrane interaction.	[87]
CZS-5	32 residues	Cruziohyla calcarifer (frog skin secretions)	X-1081 (TcI), Mg, Ds, Y (TcII) strains/epimastigotes	In vitro	X-1081 strain: 4.7 ± 1.0 μM	Erythrocytes: CC_50_ = 237.6 ± 15.2 μM; SI: 50.3	Multitarget parasite damage involving toroidal pore-mediated membrane disruption, DNA leakage, ROS increase, and energy metabolism impairment.	[88]
CZS-11	27 residues	Cruziohyla calcarifer (frog skin secretions)	X-1081 strain/epimastigotes	In vitro	12.7 ± 2.9 μM	Erythrocytes: CC_50_ = 567.5 ± 44.9 μM; SI: 44.7	Toroidal pore-mediated membrane damage supported by molecular dynamics simulations.	[88]
Bacteriocin AS-48	70 residues	*Enterococcus faecalis*	Arequipa, SN3, Tulahuen strains/epimastigotes, trypomastigotes, and amastigotes	In vitro	Epimastigotes: 0.76–1.16 μM; Amastigotes: 0.99–6.81 μM; Blood trypomastigotes: 0.11–0.19 μM	Vero cells: CC_50_ = 93.06 ± 5.67 μM; SI: 47.0–846.0	ROS-mediated mitochondrial depolarization and bioenergetic collapse.	[89]
Human Defensin α -1	30 residues	Human leukocytes and epithelial cells	MMC 20A clone (Tulahuen strain)/trypomastigotes and amastigotes	In vitro	Concentration-dependent killing (3.7–35 μM); 35% killing in blood at 25 Μm	HeLa cell line Sublethal dose (3.7 μM) tested against human cells showed reduction in parasite binding and entry.	Pore-mediated membrane disruption associated with nuclear/kDNA fragmentation and apoptosis-like alterations.	[90]

IC_50_: half-maximal inhibitory concentration; LD_50_: median lethal dose; SI: selectivity index; CC_50_: half-maximal cytotoxic concentration.

**Table 5:** Immunomodulatory and regenerative cell-based therapies for chronic Chagas cardiomyopathy.

Cell-Based Strategy	Cell Source/Intervention	Experimental or Clinical Model	Main Therapeutic Effects Reported	Proposed Mechanism of Action	Translational Limitation	Reference
Bone marrow cell therapy	Mesenchymal cells from bone marrow	Murine model of acute Chagas disease (CD1 mice infected with Brazil strain *T. cruzi*)	Reduced right ventricular dilation	Predominantly indirect cardiac action, with limited direct myocardial incorporation, despite preferential homing to damaged cardiac tissue.	Limited tracking depth; low myocardial homing of administered cells.	[99]
Genetically modified MSC therapy	IGF-1-overexpressing MSCs (MSC_IGF-1) derived from bone marrow	Murine model of chronic Chagas disease (C57BL/6 mice infected with Colombian strain *T. cruzi*)	Decreased cardiac inflammatory infiltrates and fibrosis, accompanied by recovery of skeletal muscle myofiber area.	Immunomodulatory and pro-regenerative effects; reduced TNF-α/IFN-γ; fibrosis modulation; IGF-1-mediated skeletal muscle repair.	Lack of left ventricular dysfunction in the mouse model, limiting interpretation of functional cardiac benefit.	[100]
Adipose-derived MSC therapy	Adipose tissue-derived mesenchymal stromal cells (ASCs)	Mouse model of chagasic cardiomyopathy (CD1 mice infected with Brazil strain *T. cruzi*)	Reduced blood parasitemia, cardiac inflammatory infiltrates, tissue parasite burden, and fibrosis; prevention of right ventricular dilation.	Anti-parasite immune modulation; increased IL-10 with reduced IFN-γ/TNF-α; inhibition of parasite replication in macrophages.	Poor intravenous tolerability, with pulmonary embolism; limited clinical relevance of early treatment at 3 dpi; lack of human-like left ventricular dysfunction.	[101]
Adipose-derived Mesenchymal Stem Cells (ADSCs)	Mesenchymal stem cells derived from adipose tissue.	Experimental: Chronic chagasic mouse model.	Reduction in inflammation and fibrosis within the myocardial tissue (based on conversation history regarding this source line).	Immunomodulation and secretion of paracrine factors to stimulate tissue repair.	Murine–human biological differences; lack of standardized cell dosing and delivery methods.	[101]
Cardiac MSC therapy	Cardiac mesenchymal stem cells (CMSCs) from GFP transgenic mouse hearts	Mouse model of chronic Chagas disease (C57BL/6 mice infected with Colombian strain *T. cruzi*)	Reduced cardiac inflammatory cell infiltration	Immunomodulation via reduced TNF-α and increased TGF-β in cardiac tissue; suppression of lymphoproliferation.	Fibrotic area was not reduced; cells did not differentiate into beating cardiomyocytes in vivo or in vitro.	[102]
Genetically modified MSC therapy	G-CSF-overexpressing MSCs (MSC_G-CSF) derived from bone marrow	Murine model of chronic Chagas disease (C57BL/6 mice infected with Colombian strain *T. cruzi*)	Decreased myocardial leukocyte infiltration and fibrosis, accompanied by improved exercise capacity.	Enhanced immunomodulation via Tregs and MDSCs recruitment; increased IL-10 with reduced IFN-γ/TNF-α.	ECG abnormalities not reversed; potential G-CSF-related side effects; small animal model limitations.	[103]
Tolerogenic Dendritic Cell therapy	Tolerogenic Dendritic Cells (tDCs) generated from bone marrow (dexamethasone/LPS treatment)	Chronic Chagas disease cardiomyopathy (CCC) mouse model (C57BL/6 mice infected with Colombian strain *T. cruzi*)	Reduced heart inflammation and fibrosis; reduced gene expression of *Ifng*, *Il*12, *Col*1*a*2, and *Lgals*3	Immune modulation via induction of FoxP3+ Treg cells in the heart and spleen; increased IL-10 expression; downregulation of Galectin-3.	Not clearly reported	[104,105]
Bone marrow mononuclear cell therapy	Bone marrow mononuclear cells (BMCs)	Murine model of chronic Chagas disease (BALB/c and C57BL/6 mice infected with Colombian strain *T. cruzi*)	Reduced myocarditis (inflammatory infiltrate) and interstitial fibrosis	Massive apoptosis of host-derived myocardial inflammatory cells; possible reverse remodeling reducing wall stress.	Lack of apical aneurysm and fatal arrhythmia replication in mice; discordance between murine findings and human randomized trials.	[106,107]
Bone Marrow Mononuclear Cell Transplantation (BMCT)	Autologous bone marrow mononuclear cells; intracoronary injection (slowly over 10 min).	Clinical: Phase I open-label trial with 28 patients (NYHA class III/IV).	Significant increase in LVEF (20.1% to 28.3%), improved NYHA class (3.1 to 1.8), and increased 6 min walk distance.	Paracrine anti-apoptotic effects, immune milieu modification, and potential cardiomyocyte regeneration.	Small sample size; absence of placebo control; modest benefits requiring validation in larger trials.	[108,109]

ADSCs: adipose-derived mesenchymal stem cells; ASCs: adipose tissue-derived mesenchymal stromal cells; BMCs: bone marrow-derived cells; BMCT: bone marrow mononuclear cell transplantation; CMSCs: cardiac mesenchymal stem cells; CCC: chronic Chagas cardiomyopathy; dpi: days post-infection; ECG: electrocardiogram; FoxP3: forkhead box P3; G-CSF: granulocyte colony-stimulating factor; GFP: green fluorescent protein; IFN-γ: interferon-gamma; IGF-1: insulin-like growth factor-1; IL-10: interleukin-10; LPS: lipopolysaccharide; LVEF: left ventricular ejection fraction; MDSCs: myeloid-derived suppressor cells; MSCs: mesenchymal stem/stromal cells; MSC_G-CSF: G-CSF-overexpressing mesenchymal stem/stromal cells; MSC_IGF-1: IGF-1-overexpressing mesenchymal stem/stromal cells; tDCs: tolerogenic dendritic cells; TGF-β: transforming growth factor-beta; TNF-α: tumor necrosis factor-alpha; Tregs: regulatory T cells; NYHA: New York heart association class.

## Data Availability

No new data were created or analyzed in this study. Data sharing is not applicable to this article.

## Abbreviations

The following abbreviations are used in this manuscript:

DTUs	Discrete Typing Units
MASP	Mucin-associated surface proteins
NTR	Nitroreductases
FMN	Flavin mononucleotide
FDA	Food and Drug Administration
SOD	Superoxide dismutase
IC_50_	Half-maximal inhibitory concentration
EC_50_	Maximal effective concentration
iNOS	Inducible nitric oxide synthase
ROS	Reactive Oxygen Species
GC	Gas chromatography
GC-MS	Gas chromatography–mass spectrometry
SI	Selectivity index
CC_50_	Half-maximal cytotoxic concentration.
TC_50_	Toxic concentration 50%
LC_50_	Lethal Concentration 50%
CCC	Chronic Chagas cardiomyopathy
MSCs	Mesenchymal stem cells
ASCs	Adipose tissue-derived mesenchymal stromal cells
BMCs	Bone marrow-derived cells
mDCs	Mature myeloid dendritic cells
tDCs	Tolerogenic dendritic cells
BMCT	Bone marrow mononuclear cell transplantation
CMSCs	Cardiac mesenchymal stem cells
dpi	Days post-infection
ECG	Electrocardiogram
G-CSF	Granulocyte colony-stimulating factor
IGF-1	Insulin-like growth
LPS,	Lipopolysaccharide
LVEF	Left ventricular ejection fraction
MDSCs	Myeloid-derived suppressor cells
TGF-β,	Transforming growth factor-beta
TNF-α	Tumor necrosis factor-alpha
